# A digital twin of the Biograph Vision Quadra long axial field of view PET/CT: Monte Carlo simulation and image reconstruction framework

**DOI:** 10.1186/s40658-025-00738-3

**Published:** 2025-03-31

**Authors:** Christian M. Pommranz, Ezzat A. Elmoujarkach, Wenhong Lan, Jorge Cabello, Pia M. Linder, Hong Phuc Vo, Julia G. Mannheim, Andrea Santangelo, Maurizio Conti, Christian la Fougère, Magdalena Rafecas, Fabian P. Schmidt

**Affiliations:** 1 Department of Preclinical Imaging and Radiopharmacy, Werner Siemens Imaging Center, Eberhard Karls University Tuebingen, Roentgenweg 13, 72076 Tuebingen, Germany; 2 Institute for Astronomy and Astrophysics, Eberhard Karls University Tuebingen, Sand 1, 72076 Tuebingen, Germany; 3 Institute of Medical Engineering, University of Lübeck, Lübeck, Germany; 4 Siemens Medical Solutions USA, Inc., Knoxville, TN USA; 5 Cluster of Excellence iFIT (EXC 2180) “Image Guided and Functionally Instructed Tumor Therapies”, University of Tuebingen, Tuebingen, Germany; 6 Department of Nuclear Medicine and Clinical Molecular Imaging, University Hospital Tuebingen, Tuebingen, Germany

**Keywords:** Total-body PET, GATE, Digital twin, Monte Carlo simulation, LAFOV PET

## Abstract

**Background:**

The high sensitivity and axial coverage of large axial field of view (LAFOV) PET scanners have an unmet potential for total-body PET research. Despite these technological advances, inherent challenges to PET scans such as patient motion persist. To provide simulation-derived ground truth information, we developed a digital replica of the Biograph Vision Quadra LAFOV PET/CT scanner closely mimicking real event processing and image reconstruction.

**Material and methods:**

The framework uses a GATE model in combination with vendor-specific software prototypes for event processing and image reconstruction (e7 tools, Siemens Healthineers). The framework was validated against experimental measurements following the NEMA NU-2 2018 standard. In addition, patient-like simulations were performed with the XCAT phantom, including respiratory motion and modeled lesions of 5, 10, 20 mm size, to assess the impact of motion artefacts on PET images using a motion-free reference.

**Results:**

The simulation framework demonstrated high accuracy in replicating scanner performance in terms of image quality, contrast recovery (37 mm sphere: 86.5% and 85.5%; 28 mm: 82.6% and 82.4%; 22 mm: 78.8% and 77.7%; 17 mm: 74.9% and 74.6%; 13 mm: 67.0% and 67.9%; 10 mm: 55.5% and 64.3%), image noise (CV of 7.5% and 7.7%) and sensitivity (174.6 cps/kBq and 175.3 cps/kBq) for the simulation and experimental data, respectively. High agreement was found for the spatial resolution with a difference of 0.4 ± 0.3 mm and the NECR aligned well with a maximum deviation of 9%, particularly in the clinical activity range below 10 kBq/mL. Motion induced artefacts resulted in a quantification error at lesion level between − 12.3% and − 45.1%.

**Conclusion:**

The experimentally validated digital twin of the Biograph Vision Quadra facilitates detailed studies of realistic patient scenarios while offering unprecedented opportunities for motion correction, dosimetry, AI training, and imaging protocol optimization.

## Introduction

Long axial field of view positron emission tomography and computed tomography (LAFOV PET/CT) scanners have emerged as a cutting-edge technology, pushing the boundaries of research in total-body PET [1]. The unprecedent sensitivity of up to 176 kcps/MBq [2], high TOF performance and meter-long axial coverage enable dynamic scans of the whole body with high spatio-temporal resolution [3, 4], low dose examinations [5, 6] and fast scans [7, 8].

Despite these technological advances, PET imaging continues to face inherent challenges, such as the inevitable patient motion throughout the scan, due to which the associated artefacts degrade the quantification accuracy and diagnostic value. Advanced motion correction methods aim to mitigate these effects [9, 10]. However, in the absence of a motion-free reference as ground truth information, the residual error cannot be accurately assessed. Similarly, no ground truth information is available for image-derived dosimetry [11, 12] or for optimizing low-dose scan protocols using data sets from individual patients with different injected activities [6, 13]. Moreover, the lack of ground truth information affects the training of artificial intelligence algorithms, as they must either use approximated or pseudo ground truth data or work with real patient data that is assumed to be accurate, resulting in potential inaccuracies in the AI model performance [14].

To address these challenges, a precise digital twin of the PET/CT system and hence patient data through simulations potentially offers a solution. Such an in silico representation allows for the generation of ground truth data. In this study we developed a digital replica (“Digital Twin”) of the Biograph Vision Quadra LAFOV PET/CT scanner (Siemens Healthineers, Knoxville, TN, USA) that closely mimics real event processing and image reconstruction features.

We incorporated the Monte Carlo simulation toolkit Geant4 Application for Emission Tomography (GATE) [15–17], which has been widely used to simulate preclinical [18, 19] and clinical systems [20, 21]. GATE has also been used for other LAFOV PET scanners, such as the J-PET [22], uEXPLORER [23, 24], PennPET Explorer [25] or a scanner based on monolithic detectors [26] to study, e.g., different configurations such as sparse detector assembly or different axial lengths.

Recently, Peña-Acosta et al. simulated the Biograph Vision Quadra using GATE and a generic workflow based on open-source tools [27]. Event processing is handled by the built-in GATE digitizer, which is generic to be applicable to a variety of systems. The tuning of parameters such as dead time is conventionally performed empirically without a priori knowledge of the underlying technology and performance of the scanner. Normalization [28] and image reconstruction with the Customizable and Advanced Software for Tomographic Reconstruction (CASToR) [29] complement their generic approach. While their workflow involving generic open-source tools is appealing and flexible, in this study we aim to tailor the entire workflow specifically for the Biograph Vision Quadra to obtain highest accuracy in resembling the clinical scans.

The first step involves the simulation of events in terms of physical properties and processes, including their detection. We therefore developed a GATE mass model including the patient bed based on detailed vendor-specific information. Subsequently, after obtaining simulated GATE event data, we perform a specific modeling of procedures and parameters such as coincidence sorting, randoms estimation using the delayed window method, time resolution, assembling singles from multiple single crystal events, and energy discrimination. Finally, we use the vendor specific image reconstruction software, which is the same as employed for the real scanner. This ensures that procedures such as normalization, scatter correction and attenuation correction are completely identical to the real system.

In order to validate this framework, we performed simulations regarding image quality and quantification, spatial resolution, sensitivity and count rates following the National Electrical Manufacturers Association (NEMA) NU-2 2018 standard [30]. The outcomes were compared to experimental data from the evaluation of the Biograph Vision Quadra by [2, 31]. In addition, to demonstrate the feasibility of patient-like scanning in silico, we performed simulations using the digital twin of the Quadra together with the “extended cardiac-torso” (XCAT) phantom [32]. Furthermore, we modeled respiratory motion to assess the impact of motion artefacts qualitatively and on lesion quantification accuracy and to showcase the potential use of the digital twin to obtain ground truth information via a motion-free reference.

## Materials and methods

### Monte Carlo simulation, event processing, and image reconstruction

**Fig. 1:**
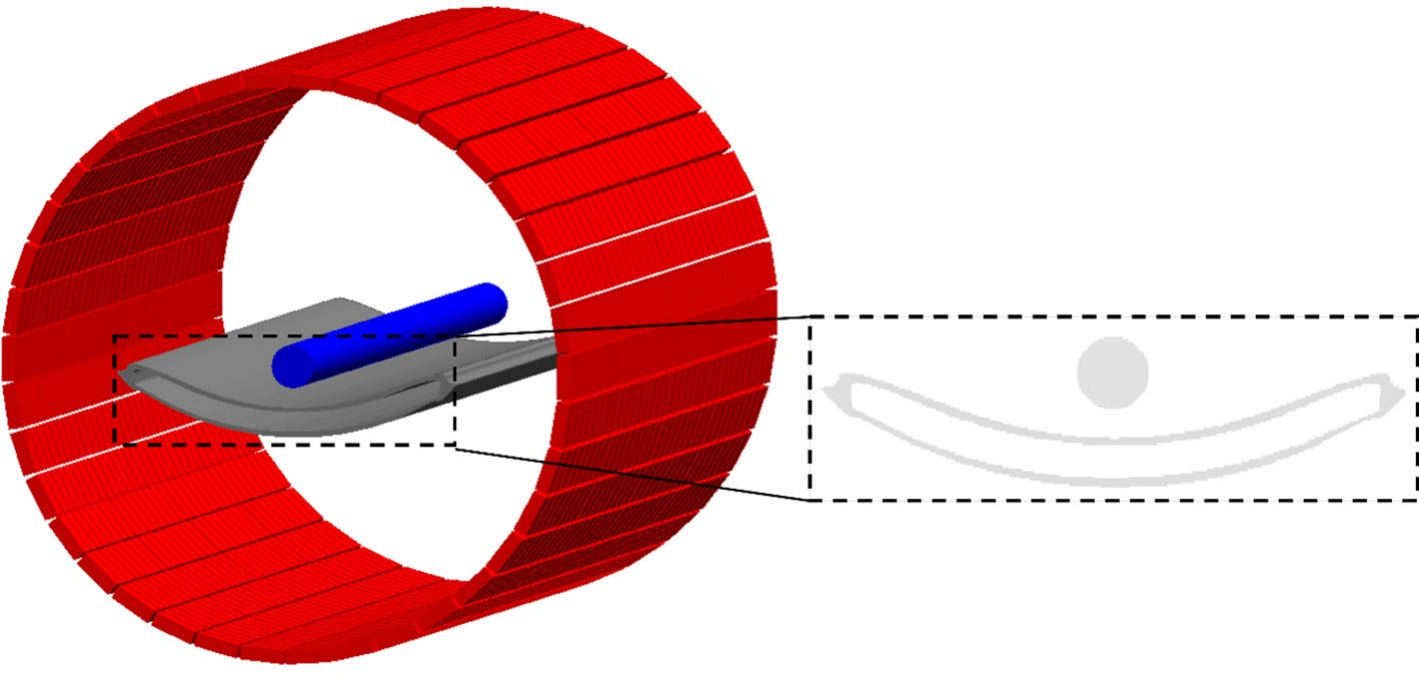
Visualization of the Biograph Vision Quadra GATE model (red). The mass model optionally includes the patient bed (grey). For visualization purposes, a cylindrical phantom (blue) is shown above the patient bed. The section highlighted in black is a zoomed-in portion of the attenuation map in the transverse view

The Monte Carlo simulations were performed with the GATE [15–17] version 9.1 using the Geometry and tracking 4 (Geant4) toolkit [33–35] version 10.7.2. A GATE mass model (Fig. 1) of the Biograph Vision LAFOV PET/CT scanner was implemented with the same specifications as the real scanner, with a bore diameter of 82 cm (crystal face-to-face) and an axial field of view (aFOV) of 106 cm. The scanner model is composed of four cylindrical segments, each containing 80 lutetium oxyorthosilicate (LSO) crystals of 3.2 × 3.2 × 20.0 mm^3^ in axial direction and 760 crystals per ring. The segments are separated by a gap of a single crystal width in size. A model of the patient bed shown in Fig. 1, which was derived from CT images of the real patient bed, was implemented as a tessellated volume composed of plastic.

The GATE simulations used the *emstandard_opt4* physics list for precise modeling of electromagnetic physics [36, 37]. The individual physical interactions (*hits)* within single crystals were added to single crystal events using the *adder* module and subsequently combined into single events using the *readout* module. An energy resolution of 9% was modeled using the *blurring* module and the events were stored in a ROOT [38] file without applying an energy cut to preserve low-energy signals for further processing. In the following step, the events were processed with an investigational software prototype root2lm (Siemens Healthineers, Knoxville, TN, USA), which accurately models the PET detectors in terms of time resolution, dead time, inter-crystal scatter and LSO background radiation as well as applies the coincidence sorter as in the real system. In contrast to the fitting of generic dead time and pile-up models to experimental data commonly performed in GATE simulation studies [39], root2lm incorporates vendor-specific models for individual detector and scanner components, representing a bottom-up modeling approach. The output of the root2lm tool is a PET listmode data file that follows the same PETLINK data format [40, 41] as used for the Biograph Vision Quadra raw data, including prompts, randoms and tags (time, singles, bed position).

Image reconstruction based on the simulated listmode data was performed using an investigational software prototype e7 tools (Siemens Healthineers, Knoxville, TN, USA), which employs identical processes, e.g. histogramming, attenuation correction, normalization, scatter simulation and correction, decay correction, frame length, and emission calibration factor, as performed by the image reconstruction for the real scanner.

**Fig. 2:**
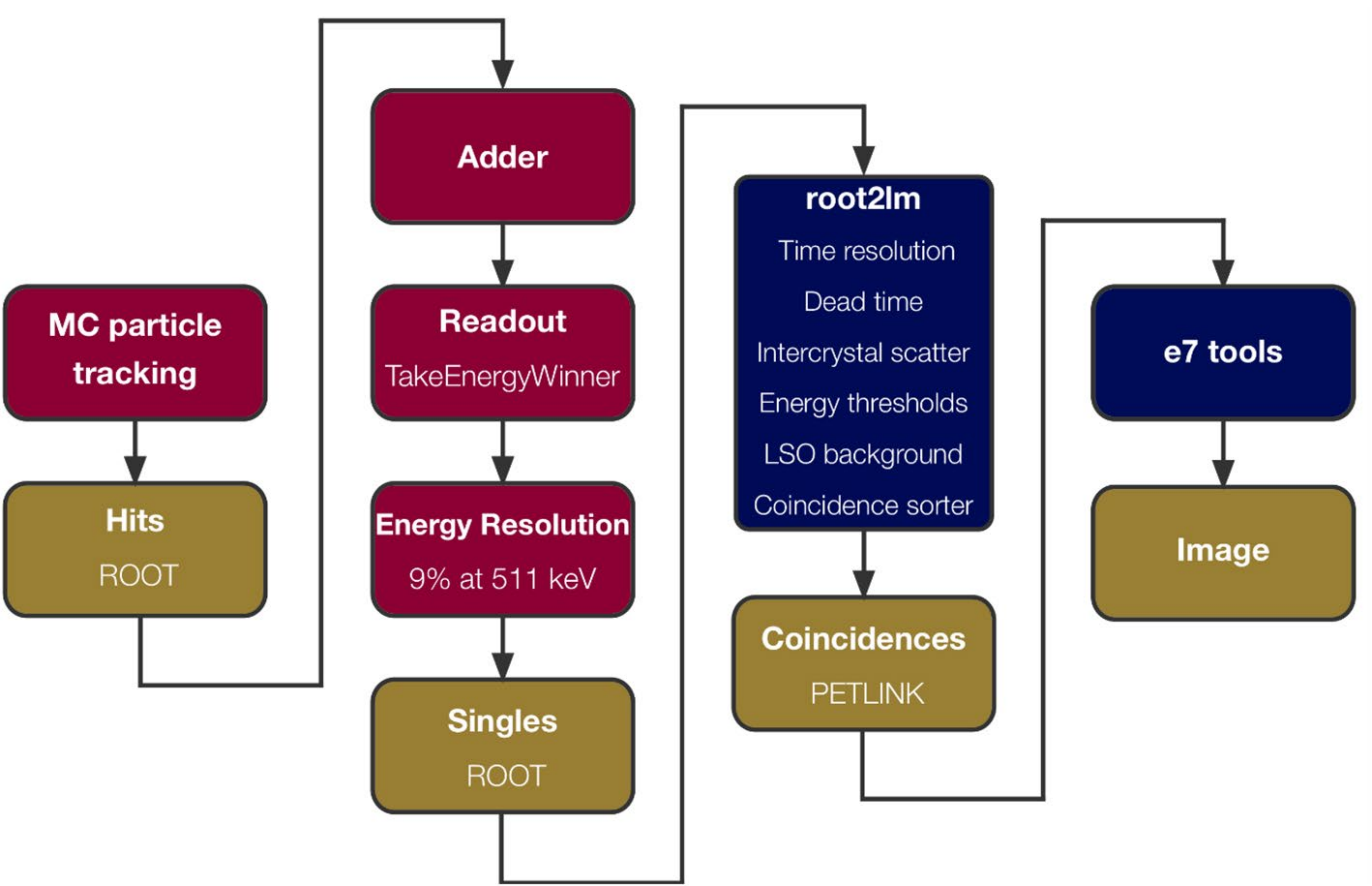
Visualization of the simulation and image reconstruction workflow. Data output is depicted in yellow, GATE in red, and software provided by Siemens Healthineers in blue

The µ-map required for attenuation correction was generated for each simulated phantom data using the GATE *MuMapActor* with a voxel size of 1.65 × 1.65 × 1.65 mm^3^. A component-based normalization file [42], which was obtained from the real scanner with a modified calibration factor, was used for normalization correction of the simulated data. Figure 2 shows an overview of the simulation and image reconstruction workflow.

### Evaluation and validation of simulations

The simulation workflow and image reconstruction framework were evaluated through different phantom simulations and analyses, which were designed based on the NEMA NU 2-2018 protocol [30], and quantitatively compared to the experimentally derived performance characteristics of the Biograph Vision Quadra reported in [2]. In addition, a visual comparison of IEC phantom images with a data set reported in [31] was done, the accuracy in quantification of activity concentrations for different phantoms was validated, and a voxelized anthropomorphic phantom was simulated to demonstrate the potential of the digital twin to investigate realistic patient scan scenarios. The Biograph Vision Quadra offers two distinct sensitivity modes, utilizing either the full maximum ring difference (MRD) of 322 or a reduced MRD of 85, corresponding to axial acceptance angles of 52° and 18°, respectively. Detailed information on the MRD configuration of the Biograph Vision Quadra can be found in [43]. At the time of the NEMA NU 2-2018 based evaluations by [2], image reconstruction with MRD322 was not yet available. While acquisitions with MRD322 were feasible, allowing to report sensitivity and count rates for both MRD85 and MRD322, image reconstruction was limited to MRD85. To ensure comparability, we matched the MRD for each evaluation as described by [2], thus all image-based analysis in this manuscript was performed based on an image reconstruction using MRD85.

#### Image quality and quantification

Image quality (IQ) was assessed using a digital phantom replicating the standard NEMA International Electrotechnical Commission (IEC) phantom [30] with an activity of 5.3 kBq/mL ^18^F for the background compartment, and sphere-to-background ratios (SBR) of 4:1 and 8:1 for all spheres. The spheres were enveloped in plastic material with a wall thickness of 1 mm. The center of the lung insert filled with air was aligned with the transaxial center of the FOV and the centers of the spheres were aligned with the axial center of the FOV. Following the NEMA NU 2-2018 protocol, a scatter phantom with 100 MBq ^18^F was placed at a distance of 50 mm from the IEC phantom. The ^18^F decay was modeled using a positron source, randomly sampling the initial positron energy from the *Fluor18* spectrum implemented in GATE.

The simulated scan time was 300 s, and the simulated listmode data were reconstructed using an Ordinary-Poisson Ordered-Subsets Expectation–Maximization (OP-OSEM) algorithm with four iterations and five subsets, point spread function (PSF) modeling, and time-of-flight (TOF) information. Images were reconstructed into a matrix of 440 × 440 × 645 voxels with a voxel size of 1.65 × 1.65 × 1.65 mm^3^.

For visual comparison with experimental derived images, the data set reported in [31] was reframed to have the same count statistics as for the simulation (background concentration of 3.30 kBq/mL and frame duration of 482 s and 3.26 kBq/mL and 488 s for SBR 4:1 and 8:1, respectively). The coefficient of variation (CV) was calculated as the ratio of the standard deviation to the mean activity concentration value of the background VOI to compare image noise. Background activity concentration was determined by the mean voxel value of a box-shaped VOI in the background (in-plane dimensions of 150 × 15 mm^2^ and an axial dimension of 160 mm).

IQ was assessed by contrast recovery coefficients (CRCs) calculated according to the formula of NEMA NU 2-2018 [30]. Activity concentrations for the spheres were determined as the mean voxel value of spherical volume-of interests (VOIs) with diameters of 10, 13, 17, 22, 28 and 37 mm for the respective spherical inserts. Furthermore, a cylindrical VOI with a diameter of 30 mm and an axial dimension of 160 mm was placed in the lung insert, and the lung residual error was calculated as the mean value of the lung VOI divided by the mean value of the background VOI.

To validate the quantification accuracy, the mean activity concentration of the background VOI for the IEC phantom was reported. In addition, to verify that the quantification is correct for different activities and geometric shapes, 300 s simulations of two different cylindrical phantoms with ^18^F were performed: a short phantom with a length of 10 cm, a diameter of 10 cm and activity concentration of 6.0 kBq/mL (5 × 5 × 5 cm^3^ VOI to determine the mean activity concentration) and a long phantom with a length of 130 cm, a diameter of 55 mm and an activity concentration of 3.0 kBq/mL (cylindrical VOI with a diameter of 35 mm diameter and an axial length of 100 cm).

#### Spatial resolution

Spatial resolution was evaluated using a digital point-like ^22^Na source modeled as a GATE ion source with an activity of 393 kBq and a diameter of 0.25 mm, which was surrounded by an acrylic cube of 10 × 10 × 10 mm^3^. Simulations of 120 s were performed at each of the six positions according to the NEMA NU 2-2018 protocol [30]: axial center FOV (0 mm) and 1/8 distance from the edge of the aFOV (397.5 mm); radial 10 mm, 100 mm and 200 mm). The positron mean free path was included in the simulations by using an ion source, including the modeling of the radioactive decay of ^22^Na, and tracking of the produced particles. Image reconstruction was performed with direct inversion Fourier transform back-projection (3D-TOFDIFT) [44] using TOF information without attenuation correction and without post-reconstruction filter. Spatial resolutions in the axial, radial and tangential directions were determined using in-house analysis tools as the full-width-at-half-maximum (FWHM) of the point spread functions according to NEMA NU 2-2018 [30].

#### Sensitivity

The system sensitivity and axial sensitivity profiles were determined with simulations of a 680 mm long line source with a diameter of 3 mm containing 4.56 MBq of ^18^F. The line source was surrounded by five 1.25 mm thick aluminum sleeves. Five 300 s long simulations were performed, with the outer sleeve removed after each simulation run. Following the NEMA NU 2-2018 protocol, the histogrammed listmode data were rebinned to single slices and the total count rate with no attenuation was determined. System sensitivities and axial sensitivity profiles (for the simulation with a single aluminum sleeve) were reported for MRD85 and MRD322.

#### Count rates: prompts, trues, randoms, scatter and noise equivalent counts

A cylindrical polyethylene phantom with 700 mm length and a diameter of 203 mm was used for count rate simulations. An 800 mm long polyethylene tube with an inner diameter of 3.2 mm containing a 700 mm long line source filled with 800 MBq ^18^F mixed with water was placed in a 6.4 mm cavity with a radial offset of 45 mm. In order to have the same conditions as for the experimental measurements, the simulation model of the patient bed was included. To mimic the radioactive decay of the phantom a total of 21 simulations were performed with activities ranging from 800 MBq down to 0.1 MBq. The minimum simulation time was 4 s and was increased toward lower activities to record more than 500,000 counts as required by the NEMA NU 2-2018 protocol [30]. Count rates for prompts, trues, scatter and noise equivalent count rate (NECR) and the scatter fraction were calculated for MRD85 and MRD322 as specified by the NEMA NU 2-2018 protocol [30]. Comparison with the measured NECR peak values reported by [2] was done by obtaining the simulated NECR values at 27.49 kBq/mL by linear interpolation.

#### Patient-like simulations with respiratory motion

To evaluate the potential of the simulation model and reconstruction framework to mimic patient scans with the Biograph Vision Quadra, the anthropomorphic “extended cardiac-torso” (XCAT) phantom [32] with a voxel size of 3.125 × 3.125 x 3.125 mm^3^ was used. A total activity of 217 MBq [^18^F]FDG was distributed according to typical mean organ-specific standardized uptake values [45, 46] and simulated using a GATE back-to-back source. A first simulation without any motion was performed for 300 s in order to mimic a typical scan duration for an [^18^F]FDG scan on the Biograph Vision Quadra at our institution. A second simulation introduced respiratory motion for the XCAT phantom, with a simplified breathing cycle composed of the following sequence, each phase lasting 60 s and with a full motion amplitude of 1.2 cm (measured at the diaphragm): full inhalation, partial exhalation (half the amplitude of full inhalation), full exhalation, partial inhalation and full inhalation. Images were reconstructed, accordingly as for the IEC phantom data set with five 60 s frames to visualize the different phases of breathing. Three spherical lesions with diameters of 5, 10 and 20 mm and an activity concentration of 10.4 kBq/mL ^18^F were modeled in the lung and liver. The impact of motion was assessed qualitatively for the 300 s motion simulation compared to the motion-free simulation and quantitatively assessed by determining activity concentrations with spherical VOIs delineating the lesions with respective diameters of 5, 10 and 20 mm.

## Results

### Image quality and quantification

**Fig. 3:**
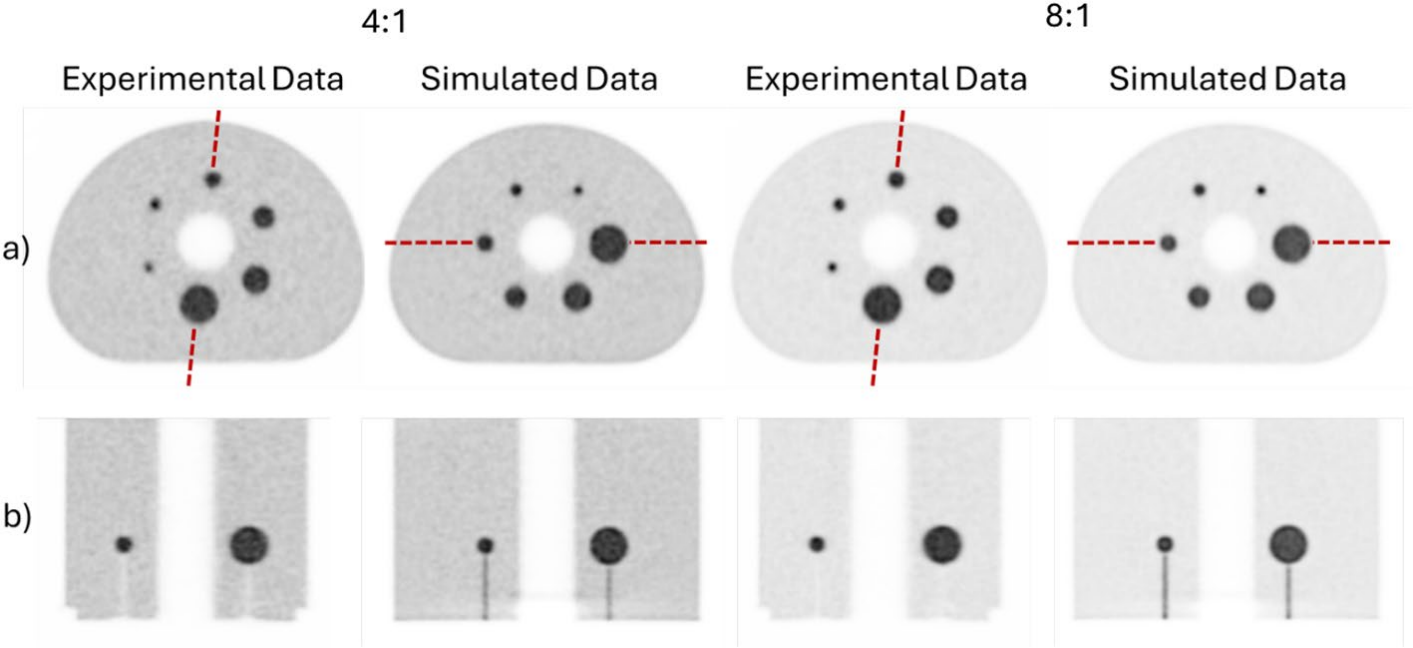
Transversal (**a**) and oblique/coronal (**b**) views of the simulated (sphere positions according to NEMA-NU 2018) and measured IEC phantom (different sphere positions and obtained from the data reported in [31]) for SBR of 4:1 and 8:1. Oblique and coronal views are obtained for the image planes indicated by the dashed lines in the transversal views

The simulation-derived images of the IEC phantom visually provide a comparable image quality to those obtained from the measured data (Fig. 3). This is confirmed by the image noise with a CV of 7.5% and 7.7% (SBR 4:1) and 7.5% and 7.6% (SBR 8:1) for the simulation and experimental data, respectively.

As shown in Table 1, a high agreement for the CRC values and the lung residual error was found for the IEC phantom simulations, with both SBRs compared to the values measured with real phantom experiments.

**Table 1:** Contrast recovery coefficients (CRC) and lung residual errors obtained with the IEC phantom with sphere-to-background ratios (SBR) of 4:1 and 8:1

Sphere Ø (mm)	SBR 4:1		SBR 8:1	
	Simulation	Experiment	Simulation	Experiment
	*CRC (%)*			
10	55.5	64.3	66.4	74.4
13	67.0	67.9	73.2	74.7
17	74.9	74.6	78.6	82.7
22	78.8	77.7	81.8	85.4
28	82.6	82.4	85.6	88.5
37	86.5	85.5	88.2	91.2
	*Lung Residual Error (%)*			
	4.7	4.8	4.8	5.1

The simulation-derived values are compared with the experimental data reported in [2]

High quantification accuracy was demonstrated by the close agreement between the activity concentrations determined from the images and the predefined values for the simulation phantoms: 5.42 kBq/mL (simulation value: 5.3 kBq/mL), 5.96 kBq/mL (6.0 kBq/mL), and 3.01 kBq/mL (3.0 kBq/mL) for the IEC phantom (SBR 4:1), and the short and long cylindrical phantoms, respectively.

### Spatial resolution

A good agreement between simulated and measured spatial resolution was obtained for all positions (Table 2) with an average absolute difference of 0.4 ± 0.3 mm (0.3 ± 0.3 mm in radial direction, 0.4 ± 0.1 mm in tangential direction, and 0.4 ± 0.3 mm in axial direction).

**Table 2:** Radial, tangential and axial spatial resolutions obtained by point source simulations at different positions and compared with experimental data reported in [2]

Position (mm)	Radial (mm)		Tangential (mm)		Axial (mm)	
	Sim	Exp	Sim	Exp	Sim	Exp
(10, 0, 0)	2.6	3.4	3.1	3.3	2.7	3.8
(100, 0, 0)	4.3	4.4	3.1	3.5	3.7	3.9
(200, 0, 0)	6.0	5.8	2.8	3.3	3.8	4.3
(10, 0, 397.5)	2.6	3.2	3.1	3.6	3.4	3.8
(100, 0, 397.5)	4.4	4.4	3.1	3.5	3.9	3.8
(200, 0, 397.5)	6.1	5.8	2.9	3.1	3.9	4.2

The largest absolute difference between simulations and measurements was 1.1 mm for the axial component and a source position at 10 mm from the center of the FOV.

### Sensitivity

For the transaxially centered line source, a high agreement was found between simulated and experimentally determined system sensitivities [2] of 82.7 cps/kBq and 82.6 cps/kBq (MRD85), and 174.6 cps/kBq and 175.3 cps/kBq (MRD322), corresponding to relative differences of 0.1% and 0.4%, respectively. For a transaxial offset of 10 cm, simulated and measured system sensitivities were 81.7 cps/kBq and 84.1 cps/kBq (MRD85), and 171.2 cps/kBq and 176.7 cps/kBq (MRD322), with the simulated sensitivities underestimating the measured sensitivities by 2.9% and 3.1%, respectively.

**Fig. 4:**
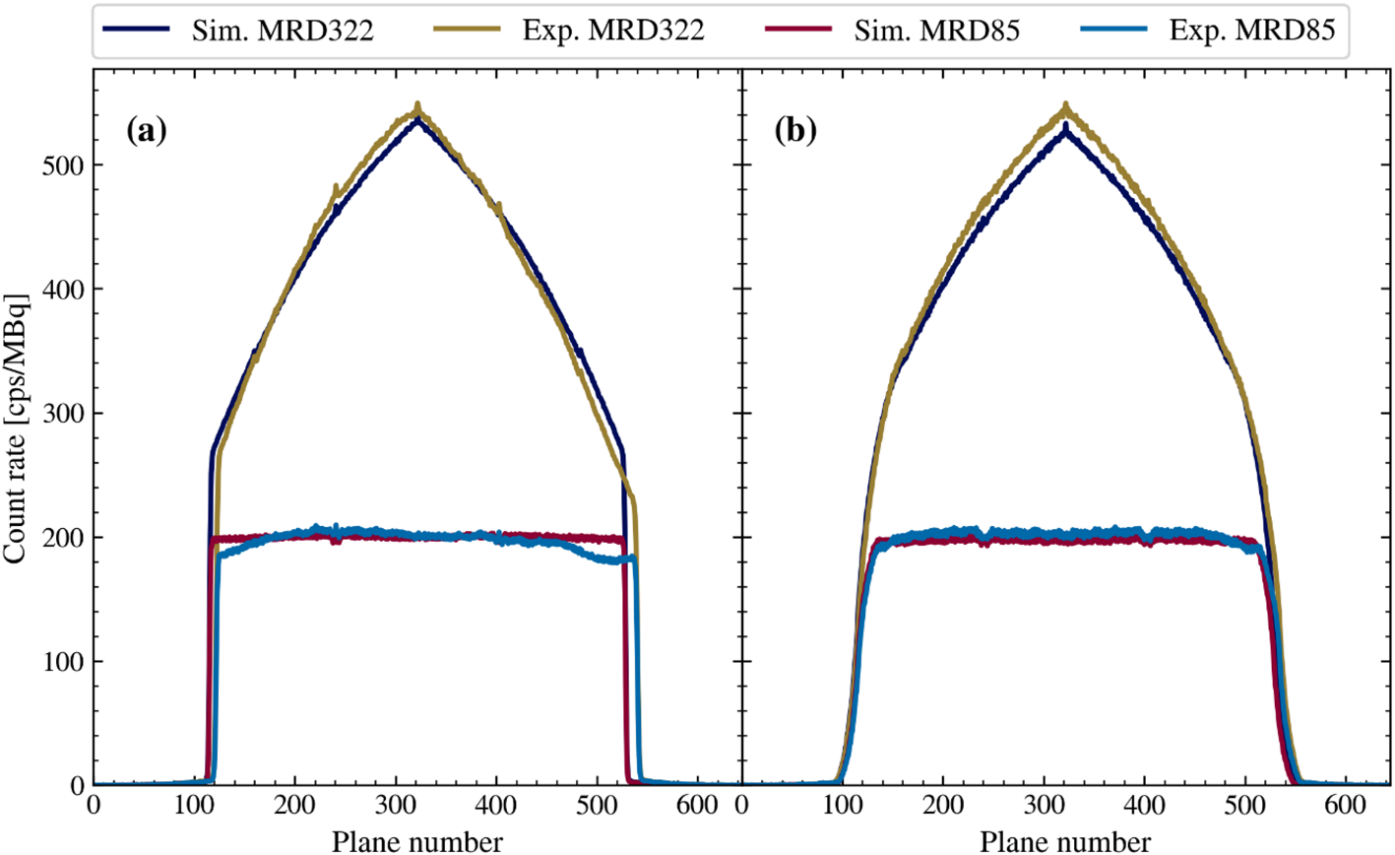
Axial sensitivity profiles obtained by simulation and measurement [2] for both MRDs with one aluminum sleeve around the line source. The line source was placed axially and transaxially centered (**a**), and with a transaxial offset of 10 cm (**b**)

The axial sensitivity profiles obtained from the experiments could be matched with the simulation model (Fig. 4).

Furthermore, the sensitivity in the central plane number 322 showed a high agreement between simulation and measurement for the transaxially centered line source (MRD85: 200.6 cps/MBq (simulation) and 200.8 cps/MBq (experiment); MRD322: 541.9 cps/MBq (simulation) and 549.4 cps/MBq (experiment)) and for the line source placed at a transaxial offset of 10 cm (MRD85: 198.5 cps/MBq (simulation) and 201.8 cps/MBq (experiment); MRD322: 533.0 cps/MBq (simulation) and 549.3 cps/MBq (experiment)).

### Count rates and scatter fraction

**Fig. 5:**
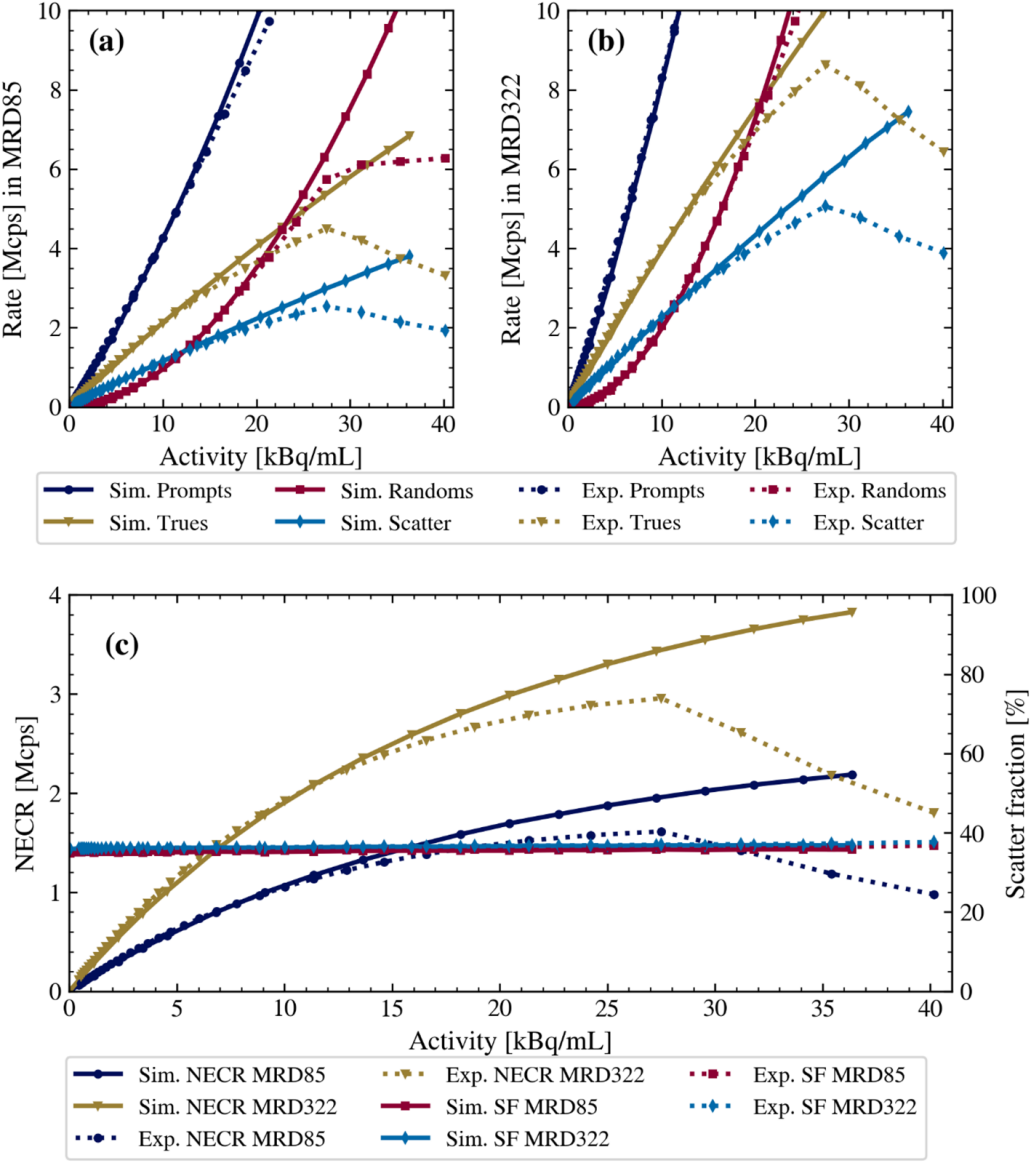
Simulated and measured count rates (experimental data reported in [2]) at different activity concentrations for MRD85 (**a**) and MRD322 (**b**). NECRs and scatter fractions for both MRDs (**c**)

The simulated count rates for trues, randoms, delays, scatters, and the NECRs are shown in Fig. 5.

In the activity range up to 10 kBq/mL, a good agreement between the simulated and the measured data was found. The relative differences in the NECRs were less than 8%/9% (MRD85/MRD322) for all measured activity concentrations below 10 kBq/mL. However, towards higher activity concentrations, the simulations overestimate the measured count rates and NECRs without the typical drop of the NECR curve for activity concentrations above the NECR peak value. At the experimentally determined NECR peak at 27.49 kBq/mL [2], the simulated NECRs were 1958 kcps (MRD85) and 3443 kcps (MRD322), an increase of 21.4% (MRD85) and 16.5% (MRD322) compared to the experimental data. Similarly, the simulated true event count rates were 19.4% (5375 kcps, MRD85) and 15.9% (10,005 kcps, MRD322) higher than the measured results. The simulations accurately reproduced the measured scatter fractions at the NECR peak position, with a scatter fraction of 36% (MRD85) and 37% (MRD322).

### Patient-like simulations with respiratory motion

**Fig. 6:**
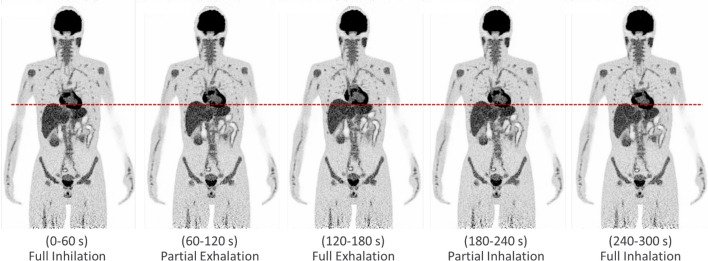
Coronal view of reconstructed images of different respiratory phases obtained by simulation with modeled breathing cycle for the XCAT phantom. The red line indicates the position of the liver dome at full inhalation

**Fig. 7:**
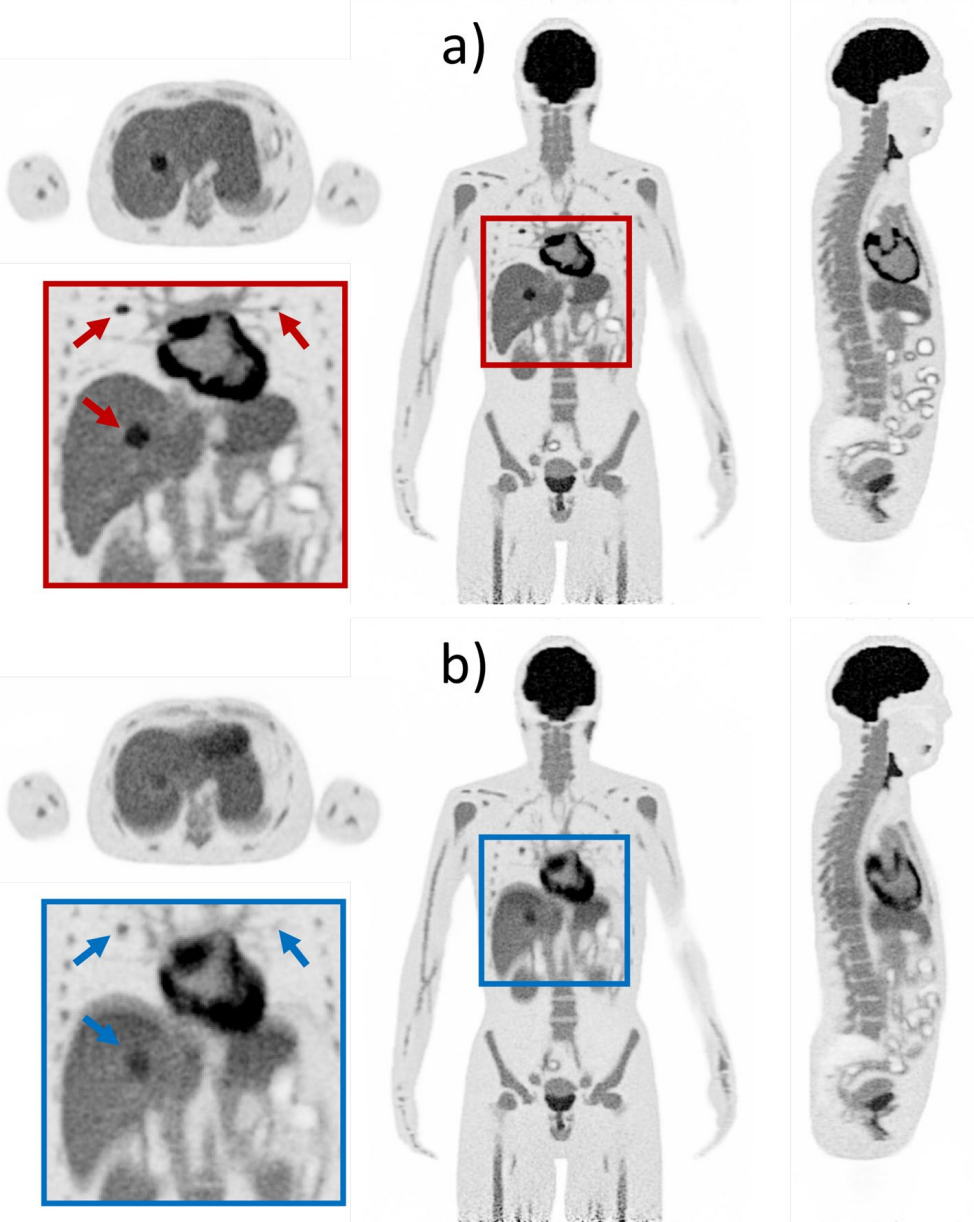
Coronal, sagittal and transversal views of the 300 s XCAT phantom simulation (**a**) without and (**b**) with respiratory motion. Partial images of the abdominal region with arrows indicating the different lesions

The motion modeled for the different respiratory phases is clearly visualized in the reconstructed images, as indicated by the different positions of the liver dome and the cyclic vertical displacement of the myocardial wall (Fig. 6). In comparison to the static reference without motion (Fig. 7a), the 300 s scan with respiratory motion produced blurring, particularly in the heart and liver, similar to that observed in PET images from real patient examinations (Fig. 7b).

The activity concentration (defined for simulation: 10.4 kBq/mL) determined for the lesions without any motion was 10.6 kBq/mL, 9.4 kBq/mL and 7.1 kBq/mL for a lesion size of 20, 10 and 5 mm, respectively. The motion induced blurring caused an underestimation of the lesion activity concentration (compared to the static reference) with 9.3 kBq/mL (− 12.3%), 6.7 kBq/mL (− 28.7%) and 3.9 kBq/mL (− 45.1%) for the 20, 10 and 5 mm lesion, respectively.

## Discussion

In this study, we first developed a workflow that closely resembles the Biograph Vision Quadra LAFOV PET/CT scanner in the digital domain using Monte Carlo simulations with GATE in combination with vendor-specific event processing and image reconstruction software. Second, to validate this framework we mimicked in silico the NEMA NU-2 2018 protocol, the standard for comparing PET scanner performance, and compared it to the experimental evaluation provided by [2] and [31]. Third, we explored the feasibility of this digital twin to simulate patient-like scenarios using the XCAT phantom.

### IQ and quantification

Visually and in terms of image noise, the images derived from the simulation workflow and from real phantom experiments exhibited comparable texture. High quantification accuracy was achieved for different phantom geometries and activity concentrations, as well as comparable contrast recovery and lung residual error to experiments with the IEC phantom.

It is noteworthy that the simulated scan time of the IEC phantom was 5 min to reduce computation time, in contrast to the 30 min for the experimental counterpart [2] to validate contrast recovery and lung residual error. It is unlikely that this is the cause for the minor deviations observed between simulated and measured CRC values, which were the highest for the smallest diameter of 10 mm, as the high sensitivity of the Biograph Vision Quadra allows for maintaining accurate contrast recovery at short scan durations. As such, it was reported by [31] that a shorter scan duration had no significant impact on the CRC as shown with an IEC phantom experiment in the Quadra with a SBR 4:1 that was 84.7%/85.1% for the 37 mm sphere (600 s/120 s) and 59.6%/59.3% for the 10 mm sphere (600 s/120 s), while in this work even a lower activity concentration was used (3.3 kBq/mL compared to 5.3 kBq/mL in our work). Moreover, we attribute the remaining differences to a bias inherent to the manual delineation of the sphere VOIs and potential air bubbles during the filling procedure. Especially the small spheres are prone to this bias, which is also observed when comparing different experiments, e.g. for the 10 mm sphere a CRC of 59.6% [31] vs. 64.3% [2].

### Spatial resolution

The spatial resolution obtained by the simulation workflow matched the experimental report, with minor deviations at some positions and a maximum deviation of 2.7 mm (simulation) vs. 3.8 mm FWHM (experiment) at position (10, 0, 0). This difference could potentially be caused by insufficient modeling of the acollinearity of annihilation photons in GATE [47]. Furthermore, the calculation method of the spatial resolution and position accuracy for real experiments can cause deviations. According to the NEMA NU-2 2018 standard the spatial resolution calculation involves a linear interpolation between two adjacent voxel values, which in our work implies a distance of 1.65 mm between neighboring interpolation points. Therefore, even small variations in the position of the point source (sub-millimeter position accuracy is not possible for a typical experimental setup) will result in variations in the FWHM. To test the impact of the source position on the determined spatial resolution, we performed additional simulations for the source position at (10, 0, 0) with sub-millimeter displacements up to 1.65 mm in axial direction. A variation of up to 0.8 mm in spatial resolution (3.5 mm) was found at the position (10, 0, 0.75), which explains the discrepancy between simulation and experimental data at this position.

### Count rates and sensitivity

The deviation between simulated and experimentally derived count rates increased towards higher activities, which is a typical behavior also observed by other groups [27, 48]. The reason for this and the lack of a peak in the NECR curve are saturation effects at the detector and system level, which are complex to model and are pronounced towards higher count rates. In particular, a limitation of GATE Monte Carlo simulations is the modeling of dead time and pile-up effects due to the independent tracking of each primary particle, which requires the modeling of inter-primary effects after particle tracking [17].

A common approach to model pile-up and dead time is using analytical models and perform simulations with varying pile-up and dead time values to obtain the best fit between experimental and simulated singles count rate data. Hereby, the optimization can either be performed to fit the true count rates while underestimating the scatter rates or apply on the scatter rates at the expense of overestimating the trues rates. Therefore, a compromise must be found between modeling accurately both rates, as shown by [48] and [39], where the latter even removed dead time modeling from the digitizer chain to obtain an optimal scatter fraction.

In this study, we did not adopt this approach of deriving dead time and pile-up values through fits to experimental data. Instead, these effects were modeled by applying a paralysable model to different components of the analog electronics, in the same way the acquisition system is designed.

Another notable challenge is the complexity of modeling the NECR peak and subsequent decrease in count rates at very high activity levels (Fig. 5c). The observed decline is when the count rate reaches the maximum events throughput [2], a phenomenon also reported in other scanners due to bandwidth limitations [49].

Future work focusing on more accurate modeling of pile-up, dead time and bandwidth limitations at high event rates may mitigate the reported differences in count rates; however, our primary objective was to provide a framework suitable for accurately simulating typical clinical scanning scenarios. Considering clinical activity concentrations below 10 kBq/mL, the simulations were consistent with experimental data, with a maximum deviation in NECR of 9% for MRD322. Similar behavior for higher and lower activity ranges was reported by [48] for a GATE model of the Discovery MI 4-ring PET/CT scanner with 33.1% and 16.5% agreement over the entire range and within the clinical activity range, respectively.

The high agreement for clinical activity concentrations was also confirmed by the results obtained with the IEC phantom (5.3 kBq/mL) and the sensitivity evaluations (4.56 MBq). Here, the sensitivity profiles and the sensitivity were consistent with the experiment with 82.7 cps/kBq/82.6 cps/kBq (simulation/experiment) and 174.6 cps/kBq/175.3 cps/kBq (simulation/experiment) for MRD85 and MRD322, respectively.

### Specific vs. generic workflow

Often, scanner models and their event processing workflow are implemented using the GATE built-in digitizer chain using generic models applicable to a variety of systems [27, 39, 50]. In our work, the vendor specific software root2lm allowed a more specific modeling of procedures and parameters such as the coincidence sorting, time resolution, assembling singles from multiple single crystal events, and energy discrimination for a closer representation of the real scanner. In GATE simulation studies, the parameters of generic models are typically obtained by fitting simulation data to experimental data (top-down approach) [39, 48]. In contrast, the root2lm tool used in this work represents a bottom-up approach implementing vendor-specific models and parameters for individual detector and scanner components. This approach results in a more realistic description of the PET scanner and allows for studying the effects of individual components on the scanner response. However, this approach leads to more complex count rate simulations at higher activities requiring precise modeling of saturation effects on the detector and system levels.

Another common approach is to use generic software for image reconstruction, such as CASToR [29] or STIR [51], which offer great flexibility and established interfaces to GATE simulations. However, in order to fully replicate the image reconstruction of the real scanner, the use of the vendor specific image reconstruction software e7 tools is the predestined method to ensure that procedures such as normalization, scatter correction, random estimation and attenuation correction, are fully identical to the real system.

While system specific modeling inherently limits the portability to other systems, it is noteworthy that the simulation and processing pipeline can be easily transferred to the Biograph Vision (Siemens Healthineers, Knoxville, TN, USA) PET/CT scanner, sharing the same detector technology, event processing and system design as the Quadra, except for a reduced aFOV of 25.4 cm.

### Patient-like simulations

In summary, from the evaluations of simulations with in silico counterparts of real phantoms, we report with this framework an accurate representation in the digital domain (“digital twin”) of the Biograph Vision Quadra.

The great potential of this digital twin could be exploited by using it with patient-like phantoms, which can be either derived from individual real patient scans or based on more generic models such as the XCAT phantom used in this work. An advantage of the latter is the ability to model whole-body, cardiac and respiratory motion. As shown in this work, this could be used to assess the impact of motion-induced artifacts on lesion quantification by comparison with a motion-free reference, which is impossible to obtain for real patient scans. In particular, LAFOV PET/CT scanners such as the Quadra are prone to motion artifacts [9], given their high spatio-temporal resolution and long axial coverage, including multiple regions of the body that follow different irregular motion patterns. As a first step, these simulations can foster the understanding of motion artifacts at the whole-body image level for the Biograph Vision Quadra. In a second step, we plan to use the digital twin as a benchmark for motion correction methods. Hereby, modeling of different motion scenarios, uptake patterns, lesion size and position, scan durations, all reflecting a variety of realistic patient scan scenarios, could be used to quantify the residual error of motion correction or to predetermine use cases for one method over the other. In a third step, we envision using simulation-derived information about the limitations of a particular correction method to improve the underlying algorithm, ultimately advancing motion correction for real patient scans.

Furthermore, realistic voxelized phantoms derived from patient PET and CT images could be used to establish simulation-derived ground truth information for dosimetric approaches. Specifically, we aim to optimize the dosimetry derived from Y-90 imaging of patients that underwent selective internal radiation therapy and were scanned on the Quadra at our institution [11]. Here, the patient images are used to create phantoms that will be (1) used to determine an accurate dose deposition (ground truth) using conventional Monte Carlo simulations, (2) processed by the framework proposed in this work to obtain PET images as if the phantom had been scanned on the real system. These images are then used to apply different dose estimation methods, e.g. kernel-based, semi/full Monte Carlo simulation [12]. Subsequently, a comparison with the ground truth obtained by (1) assists to identify how the dose estimation method or the generation of the PET image (e.g. number of iterations, filter, matrix size, scan time) needs to be modified to optimize the dosimetry.

Scatter correction is especially challenging in Y-90 imaging due to Bremsstrahlung, but it is a common issue for all isotopes used in PET, affecting quantification accuracy. Of note, although the simulation model provides ground truth information on scattered events, this data was not utilized in the present work to closely mirror the processing methods employed by the real system. Consequently, validating and improving the vendor-specific scatter simulation and correction using the simulation-derived ground truth from this model represents an interesting area for future research.

Another appealing application for realistic patient phantoms in combination with the proposed framework is the evaluation of true low dose examinations. Conventionally, this is done by rebinning PET listmode data to mimic lower dose examinations by reducing scan times. However, this presents a simplification as motion artifacts (less prominent at shorter scan durations) and event statistics (e.g., random rate and crystal background radiation rate, dead time) are not preserved. The ability to perform true low dose examinations in silico can be used to determine low dose scan protocols without sacrificing diagnostic accuracy, as well as to train AI-based methods to recover full dose image quality from low dose images [52].

## Conclusion

In this study, a comprehensive Monte Carlo simulation and image reconstruction framework was developed to replicate the Biograph Vision Quadra LAFOV PET/CT scanner in the digital domain. Validation against experimental phantom scans according to the NEMA NU-2 2018 attested reproducible image quality metrics, quantification, spatial resolution, system sensitivity and count rates for clinically relevant activities. This digital twin serves as an invaluable tool for simulating realistic patient-like scenarios, enabling detailed studies to optimize imaging protocols, offering accurate training data for image denoising, and providing ground truth information for dosimetry and motion correction. To this end, we have demonstrated the feasibility of this framework to study the impact of motion-induced artifacts with a patient-like phantom compared to a true motion-free reference.

## Data Availability

The datasets used and/or analyzed during the current study are available from the corresponding author on reasonable request. The macro files that were used in this study could be made available upon reasonable request and approval by Siemens Healthineers.
